# Traitement chirurgical par plaque à compression des fractures de Galeazzi chez l'adulte: à propos de 28 cas

**DOI:** 10.11604/pamj.2013.16.61.2856

**Published:** 2013-10-21

**Authors:** Khalid Ibn El Kadi, Mounir Benabid, Sarr Saliou, Said Zizah, Amine Mezzani, Kamal Lahrach, Amine Marzouki, Fawzi Boutayeb

**Affiliations:** 1 Service de chirurgie orthopédique et traumatologique (A), Pr F.Boutayeb, centre hospitalier universitaire Hassan II de Fès, Maroc

**Keywords:** Fracture de Galleazzi, plaque vissée, Galleazzi Fracture, bone plate

## Abstract

La fracture de Galeazzi associe une fracture diaphysaire du radius ou des deux os de l'avant bras à une luxation de l'articulation radio ulnaire distale. Décrite en 1934, sa fréquence varie chez l'adulte entre 2,7% et 6,8% de l'ensemble des fractures de l'avant bras. Le traitement admis de façon consensuel chez l'adulte est chirurgical reposant sur une ostéosynthèse stable par une plaque vissée de compression dynamique associée ou non à un embrochage de la radio ulnaire distale. Nous rapportons dans notre étude les résultats cliniques de 28 patients colligés au service de traumatologie et orthopédie A du CHU Hassan II de Fès sur une période de 06 ans. L’âge moyen de nos patients était de 30 ans avec prédominance masculine de 90%; tous nos patients ont présenté un traumatisme de poignet lors d'un accident de sport. Le côté droit était atteint dans 75% des cas. Le bilan radiologique objectiva une fracture diaphysaire du raduis associée à une luxation radio ulnaire distale; nous avons adopté la classification de de Mansat. Le traitement a consisté en une synthèse par une plaque vissée dynamique associée à un embrochage transversal chez six patients qui ont présenté une instabilité de la radio ulnaire distale. L'immobilisation par attelle plâtrée postérieure BABP était de mise. Après un recul de 36 mois, nos résultats ont été très satisfaisants suivant le score de Mestdagh, avec bonne récupération de la mobilité du poignet et reprise de toute activité sportive.

## Introduction

La fracture luxation de Galeazzi associe une fracture du radius à une luxation de l'articulation radio ulnaire distale (RUD) ¬ [1, 2]. Cette fracture prend son nom du chirurgien italien RICCARDO GALEAZZI qui l'a rebaptisé en 1934, mais d'abord décrite par Sir Astley COOPER en 1822 selon Voigt et Lill [3]. Ces fractures dont le mécanisme typique est défini comme une chute sur une main tendue dans une hyperpronation [4] se produisent avec une incidence chez les adultes de 2,7% à 6,8% de l'ensemble des fractures de l'avant bras [3, 5]. Il s'agit d'une lésion instable dominée par la méconnaissance de la luxation de la RUD au profit d'une fracture isolée du radius qui n'existe pas en principe [1]. Le traitement consensuel de ces fractures repose sur une ostéosynthèse solide et rigide associée à une immobilisation de la l'articulation radio ulnaire distale. Nous avons réalisé une étude rétrospective dans les fractures de Galleazzi à propos de 28 cas dont tous nos malades ont bénéficié d'une ostéosynthèse par plaque vissée Dynamic Compression Plate (DCP) dont les résultats cliniques sont satisfaisants avec un recul de 36mois. Notre travail à pour but essentiel de définir les modalités chirurgicales de la prise en charge de ces fractures ainsi de comparer l’évolution clinique de nos malades traités par foyer ouvert par rapport aux autres moyens chirurgicaux.

## Méthodes

Notre étude est rétrospective portant sur 28 cas de fractures de Galeazzi traitées au sein du service de traumatologie-orthopédie A du CHU HASSAN II de Fès (MAROC) durant une période de six ans allant du mois de Janvier 2006 au mois de décembre 2011. L’âge moyen de nos patients était de 30 ans (19-36ans) avec prédominance masculine (25 sur 3 femmes). Des traumatismes violents suite à des accidents de sport ont provoqué la lésion (chutes à bicyclette, football). Toutes les fractures étaient fermées, le côté droit atteint dans 21cas et le côté gauche dans sept cas (côté dominant). L'examen clinique initial retrouvait attitude du traumatisé du membre supérieur avec déformation de l'avant bras et du poignet et une impotence fonctionnelle totale du membre supérieure.la recherche clinique d'une luxation de la radio ulnaire distale devant une fracture isolée du radius et ou des deux os de l'avant bras à n'importe quel niveau de l'arbre diaphysaire était systématique. Un bilan radiologique fait de radiographie standard l'avant bras face et profil prenant les articulations du coude et du poignet. Sur le plan anatomopathologique et selon la classification de Mansat et al [6] (Tableau 1), les fractures de notre série étaient réparties comme suit: entorses: six cas; sub-luxations: neuf cas; luxations: dix cas; equivalents Galleazzi: trois cas.

**Tableau 1:** La répartition des fractures de la série selon classification de Mansat et al.

Classification	Degré de la lésion	Nombre de cas
**Type 1**	Stable avec lésion isolée du ligament triangulaire	6 patients
**Type 2**	Instable avec subluxation de la tête.	8 patients
**Type 3**	Instable avec une luxation palmaire ou plus souvent dorsale de la tête.	10 patients
**Type 4**	Equivalent de Galeazzi avec une fracture de l'apophyse styloïde	4 patients

**Figure 1:**
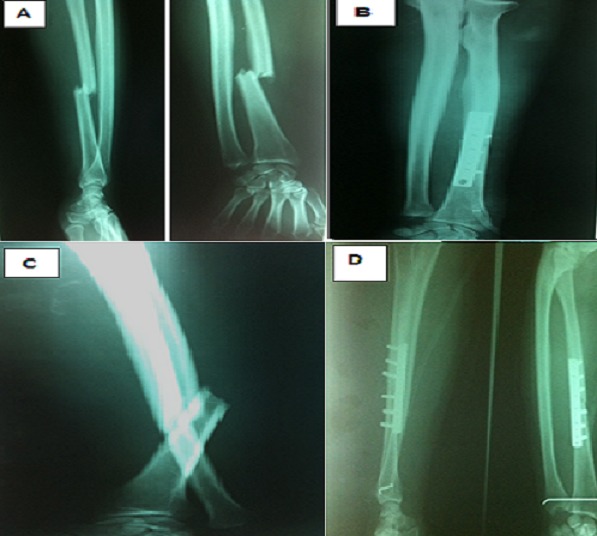
A) radiographie de face et de profil montrant une fracture de Galeazzi type 1. B) radiographie postopératoire de contrôle synthèse par plaque vissée DCP 8 trous; C) radiographie montrant une fracture de Galeazzi type 3 dorsale; D) radiographie de contrôle après synthèse par plaque vissée DCP 07 trous et embrochage de la RUD

**Figure 2:**
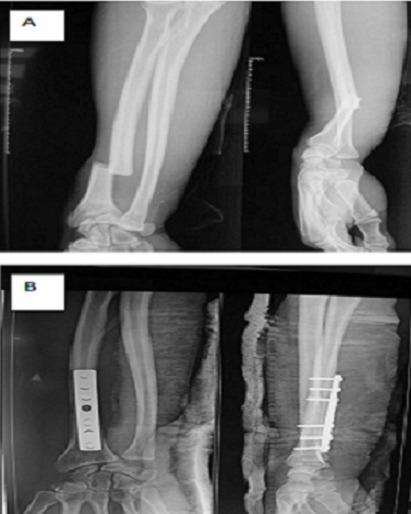
A) Radiographies du poignet (face et profil) montrant une fracture de Galeazzi type 3; B) Radiographies du poignet (face et profil) après ostéosynthèse de la fracture du radius par plaque vissée DCP 07 trous avec réduction spontanée de la luxation radio-ulnaire distale

**Figure 3:**
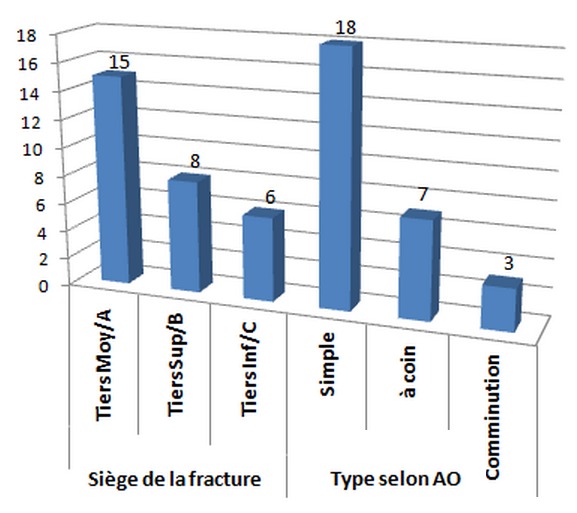
Tableau récapitulatif du siège et du type de fracture selon la classification de Müller de l'AO

Les patients ont été opérés après un délai moyen de 24 heures (huit à 48 heures), sous anesthésie générale ou locorégionale. Ils ont bénéficié d'une ostéosynthèse par une plaque dynamique de compression pour un montage solide dont la taille de la plaque dépendait exclusivement de la fracture et du degré de comminution. Le contrôle radiologique post opératoire a objectivé une réduction du foyer de fracture avec une bonne congruence articulaire radio-ulnaire distale après (Figure 1). Il s'en suit d'une immobilisation par une attelle plâtrée BAPB (brachio-antébrachio-palmaire). Chez 07 de nos patient qui présentés des luxations de la RUD très instable on a eu recours à un embrochage transversal par broche de Kirshner 18 /10de l'articulation distale radio-ulnaire suivie d'une immobilisation par attelle BABP en position neutre. Les autres 21 patients ont bénéficié en postopératoire immédiate d'une immobilisation en supination (Figure 2). La luxation dorsale de l'ulna dominée dans notre série avec 90% des patients et 10% palmaire. Les fractures du radius sont classées selon la classification de Müller de l'AO réparties en simples dans 18 cas, à coins dans 7 cas et avec une comminution dans 3 cas (Figure 3). Le siège était le tiers moyen dans 15 cas, supérieur dans 8 cas et distal dans 5 cas.

L'ablation de la broche ulno-radiale et des contentions plâtrées était faite en moyenne à 28 jours (extrêmes de 21 à 41 jours). Dès lors, une rééducation a été entreprise pour une durée minimum de huit semaines. La consolidation a été obtenue au bout de 10 semaines en moyenne, a noté que 03 patients sont perdu de vu durant le suivi clinique d'où un total de 25 patients évalué.

## Résultats

Tous nos patients ont été revus avec un recul moyen de 36 mois. Nous avons adopté la cotation fonctionnelle et clinique de Mestdagh [7] (Tableau 2) pour évaluer nos résultats. Cette cotation tient compte de quatre critères, dont des données subjectives fournies par l'interrogatoire (douleur) et des données objectives fournies par l'examen clinique (la force de préhension, saillie de la tête ulnaire et la mobilité du poignet et de la pronosupination). Nos résultats cliniques et fonctionnels globaux selon la cotation de Mestdagh étaient en moyenne de 7 sur 20 points (extrêmes de six à 13), avec 19 très bien, 5 bien et un passable résultat. Aucun mauvais résultat n'a été noté.

**Tableau 2:** Critères d’évaluation clinique selon la cotation de Mestdagh et al.

Notes	Points
	
**Douleur**	
Douleur absente ou rare, météorologique	1
Douleur à l'effort important mais inconstant	2
Douleur à tous les mouvements du poignet mais sans limitation de l'activité	3
Douleur importante avec limitation de l'activité	4
	
**Force de préhension comparée au côte sain**	
Identique	1
Force diminuée de ¼	2
Force diminuée de ½	3
Force diminuée de ¾ ou plus	4
	
**Saillie de la tête ulnaire comparée au côte sain**	
Tête ulnaire de morphologiquement normale	1
Saillie nette	2
Saillie plus nette	3
	
**Mobilite comparée au côte sain**	
**Flexion/extension**	
Normale	1
Flexion/extension déficitaire de 20°	2
Flexion/extension déficitaire de plus de 20°	3
**Inclinaison radio-ulnaire**	
Normale	1
Inclinaison radio-ulnaire déficitaire de 20°	2
Inclinaison radio-ulnaire déficitaire de plus de 20°	3
**Pronosupination**	
Normale	1
Déficitaire de 20°	2
Déficitaire de plus de 20°	3
**L'addition des côtés donne un total de 6 à 20 points**.	**Total = 20**

Les résultats: très bien = (six à sept points); bien = (huit à 12 points); passables = (13 à 16 points); mauvais = (17 à 20 points).

Au total du nombre de 25 patients évalués au bout de l’étude 20 étaient très satisfait du résultat avec une reprise de l'activité sportive antérieure sans gène fonctionnel et les 05 autres pour la plupart non satisfait à 100% à cause d'une cicatrice inesthétique ceci étant vu sur 02 femmes et 03 autres gènes par des troubles de la sensibilité. L'analyse des complications a objectivé un cas de neuroalgodystrophie ayant bien répondu au traitement, un cas de sepsis superficiel en regard des broches qui a été bien jugulé sous traitement antibiotique et soins locaux. On n'a pas noté de cas de pseudarthrose, ni de cal vicieux, ni de synostose radio ulnaire au dernier recul.

## Discussion

Le diagnostic de la fracture de Galleazzi n'est pas difficile mais souvent méconnu. Si la fracture de l'arbre diaphysaire du radius est évidente, la luxation de la radio ulnaire distale peut être méconnue [2, 6, 8]. Dans notre série cette lésion était recherchée systématiquement devant une fracture isolée du radius reposant sur la clinique et la radiologie.

La suspicion clinique est confirmée par la radiographie avec une modification de l'index radio ulnaire distale et les images devraient inclure une radiographie latérale vraie fait avec l′avant-bras en rotation neutre. Toute déviation de> 10° à partir d′une image fidèle latérale permettra de réduire considérablement la précision de l′examen. La variance ulnaire doit être mesurée et comparée à celle sur le côté opposé sur les radiographies faites avec l′avant-bras en rotation neutre et l′épaule et le coude à 90° de flexion avec le faisceau de rayons X dirigé d′arrière en avant. Les signes radiographiques de lésion de l′articulation radio-ulnaire distale comprennent une fracture à la base de la styloïde ulnaire, l′élargissement de l′espace radio-ulnaire distale commune vu sur la radiographie postéro-antérieure, de l′angulation radiale dorsale >20°, et supérieur à 5 mm de déplacement proximal de la partie distale du radius [9].

La tomodensitométrie est la technique de choix pour évaluer la congruence de l′articulation radio-ulnaire distale, mais la même information peut être obtenue avec l′imagerie par résonance magnétique. Il existe plusieurs méthodes pour évaluer la subluxation de l′articulation radio-ulnaire distale, y compris la méthode décrite par Mino et coll [10, 11], la congruence méthode [12], l′épicentre méthode[12], et la RUR (ratio de la radio-ulnaire) méthode[13]. L′imagerie par résonance magnétique est utile pour identifier les lésions du fibrocartilage triangulaire, mais sa spécificité et la sensibilité varie [14].

Chez les adultes, les fractures de Galleazzi exigent habituellement un traitement chirurgical. De nombreux auteurs ont déclaré un taux considérable de déplacements secondaires et pseudarthroses en cas d′un traitement conservateur [8, 15–17]. La série de Hughson [15] et al en 1957 en est une preuve avec un taux de 92% de mauvais résultats.

La réduction par foyer ouvert avec fixation de la fracture radiale en utilisant des plaques et des vis de compression, et d'un embrochage radio ulnaire en cas d'instabilité, et l′immobilisation par attelle à conduit à de bons résultats fonctionnels et est donc recommandé comme traitement de choix chez les patients adultes [8, 16, 17]. Ce traitement reconnu par ses résultats cliniques a était instauré chez tous nos patients, chose qui peut expliquer entre autres les résultats très bons de notre série.

La limite de notre traitement repose sur le nombre limité de patient ayant était sélectionné et d'un manque de stratégie sur l'immobilisation par attelle car certains auteurs l'impute d'une laxité résiduelle qui peut persistée pouvant être en rapport avec la fonte musculaire [3, 8].

Cependant on constate que si le traitement consensuel de cette lésion est la plaque de compression car permettant de rétablir la courbure pronatrice du radius qui est l'une des conditions nécessaire de la pronosupination [1, 8, 18] car certains auteurs admettent une réduction spontané de la luxation [8, 19]. Cette instabilité de la radio ulnaire distale peut faire appel un brochage en position de fonction et la main se met en inclinaison radiale assurant une meilleure stabilité et le carré pronateur joue un rôle actif de coaptation de la radio ulnaire distale [1, 17, 19].

Certaines complications décrites par les auteurs comme Moore et al [18] avec 39% sur 82 fractures étudiées réparties en deux ostéites, sept lésions iatrogènes peropératoires du rameau superficiel du nerf radial et des défauts techniques de montage avec leurs corollaires ne sont vu de façon sporadique concernant dans notre série 3 atteintes sensitives persistantes.

Toutefois tous les auteurs à l'unanimité sont adeptes à ce traitement [1, 7, 20–23].

A ce traitement chirurgical s'y ajoute d'autres méthodes d'ostéosynthèse. L'embrochage à foyer fermé par le clou de Rush avait donné spécialement satisfaction à Mikic [8]. En revanche, avec les broches de Kirschner, le résultat n’était pas satisfaisant chez huit patients. Deux de ces huit patients avaient développé une pseudarthrose. Ceci peut être du une manque de rigidité du montage. Ce même concept de broche à était adopté par ROTHE et Al [17] qui ont obtenu deux cas de pseudarthrose sur quatre embrochage dans une série de 25 patients. Ceci montre une place limité de l'embrochage dans les fractures de Galleazzi ou de Montéggia car étant des fractures complexe.

Une série de l’équipe de V.Dansokho et al [24] à montré que l'association d'un brochage de la radio ulnaire distale en plus des broches centromédullaire permettait d’étendre les indications dans les fractures de Galleazzi et l’équipe de MONDOR [25] corrobore cette théorie par la conservation de l'hématome péri-fracturaire et l’élasticité du montage permettant d'obtenir une consolidation rapide et d'autoriser une kinésithérapie précoce.

Considérant la gravité de la plupart des fractures étudiées d'autant plus que le risque d'instabilité est accru en cas de méconnaissance initiale des lésions, les résultats finaux très satisfaisants (bons résultats cliniques, anatomiques et fonctionnels) et l'absence de complications sévères, les auteurs concluent que le traitement chirurgical par plaques à compression donne de bons résultats comparés à ceux de l'embrochage centromédullaire. La rigidité et la stabilité du montage du radius et l'immobilisation plâtrée complémentaire systématique sont les conditions de réussite du traitement.

## Conclusion

La fracture de Galeazzi reste une entité peu fréquente mais sous diagnostiquée dans certains cas. Un examen clinique complet à la recherche d'une instabilité de la radio ulnaire distale est systématique dans les fractures du radius isolé dont la méconnaissance peut être le fruit d'instabilité et de douleur. Le traitement chirurgical reposant sur la réduction anatomique et l'ostéosynthèse stable par plaque à compression permet dans la plupart des cas la réduction simultanée de la luxation, l′embrochage radio cubital systématique en vue d′éviter la reproduction de la luxation sous plâtre ne semble pas indispensable, cependant une évaluation clinique et radiologique de la stabilité de l'articulation RUD doit être systématique Après la fixation. Le pronostic reste favorable si le traitement est adéquat et aucune complication ne doit s'inviter dans l’évolution.
